# Activation of the Dimer of 3, 4‐dimethylphenol Production From Marine *Streptomyces* sp. FJNU027 Under Oligotrophic Condition

**DOI:** 10.1002/mbo3.70191

**Published:** 2025-12-01

**Authors:** Feifei Wang, Huimin Yuan, Cuie Bai, Haiyan Li, Li Xu, Lingjun Yu, Lianzhong Luo, Yongbiao Zheng

**Affiliations:** ^1^ School of Life Sciences Fujian Normal University Fuzhou China; ^2^ Engineering Research Center of Marine Biopharmaceutical Resource Xiamen Medical College Xiamen China

**Keywords:** activation, oligotrophic medium, secondary metabolism, *Streptomyces*

## Abstract

Activating cryptic secondary metabolic gene clusters is a critical area of research in *Streptomyces*, and the cultivation‐based approach is one of effective ways to induce the expression of cryptic gene clusters. In this study, the oligotrophic medium and modified Gauze's medium were used to culture the marine *Streptomyces* sp. FJNU027 strain, and a unique secondary metabolite in oligotrophic culture was found by HPLC assay when compared with the modified Gauze's culture. Then the differential product was isolated through large‐scale fermentation, solvent extraction, column chromatography over Sephadex LH‐20, and HPLC preparation. The pure differential product was analyzed by NMR and LC‐MS, and identified as 4,4′,5,5′‐tetramethyl‐[1,1′‐ diphenyl]‐2,2′‐diol. To elucidate the possible biosynthesis mechanism of the differential product, the transcriptome sequencing was performed. It showed the expressions of polyketide synthase gene (FZ01GL006410) and cytochrome P450 gene (FZ01GL006417) were significantly enhanced in the oligotrophic medium, and these two genes might be responsible for the biosynthesis of the differential product. This compound was reported for the first time isolated from a natural source, demonstrating a novel approach for acquiring this type of compound. The results indicate that oligotrophic culture is an effective method for modulating the secondary metabolism of *Streptomyces*.

## Graphical abstract

This study assessed the potential for secondary metabolites production of marine *Streptomyces* sp. FJNU027 under various media. It was found the FJNU027 strain produced a differential compound in oligotrophic medium. The pure compound was obtained through mass fermentation, extraction and isolation, and identified as the dimer of 3, 4‐dimethylphenol using NMR and LC‐MS analysis.

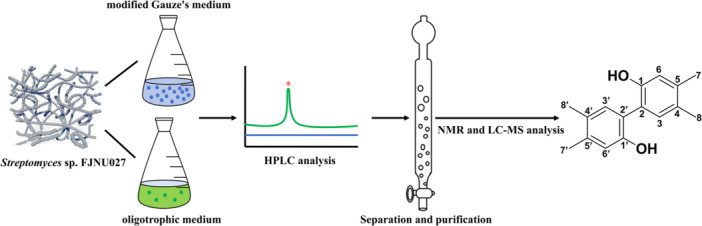

## 1 Introduction

*Streptomyces* is the main group of antibiotics‐producing microorganisms. Because of its complex morphological differentiation and metabolic network, it could produce a diverse array of active secondary metabolites (SMs) with novel structures, which had always been important lead compounds for new drug development (Thomford et al. 2018). Nowadays, marine *Streptomyces* have emerged as a promising source of bioactive SMs. The marine environments including high salt, high pressure, low temperature, and low osmosis offer unique metabolic pathways to marine *Streptomyces* which led to the biosynthesis of novel SMs with a wide spectrum of biological activities (Chen et al. 2021).

SMs are produced by their own biosynthetic gene clusters (BGCs), on average, a single *Streptomyces* strain has more than 30 BGCs in its genome through antiSMASH analysis (Lee et al. 2021). However, the majority of BGCs are cryptic which means the genes that encode biosynthetic enzymes have been identified, yet no corresponding product has been detected under standard laboratory cultivation conditions (Hoskisson and Seipke 2020). To expand the SMs library of *Streptomyces*, the activation of cryptic BGCs is imperative for researchers. Cultivation‐based approaches and molecular techniques are the main strategies to activate the large number of cryptic BGCs (Pinedo‐Rivilla et al. 2022). The first cultivation‐based strategy was named one strain‐many compounds (OSMAC) method, which posited that each microbial strain has the potential to produce multiple compounds, though only subsets of these compounds are produced under specific growth conditions (Bode et al. 2002). Therefore, variations in cultivation parameters such as medium components, cultural conditions, and addition of metal ions, chemical elicitors and epigenetic modifiers could stimulate the production of the novel SMs (Schwarz et al. 2021; Alwali and Parkinson 2023; Pillay et al. 2022). Additionally, the molecular techniques including CRISPR‐Cas9 promoter engineering, ribosome engineering, regulator manipulation, BGC reconstruction and heterologous expression are also adopted to access the cryptic BGCs (Jiang et al. 2021; Zhu et al. 2019; Li et al. 2021; Deng et al. 2024; Pait et al. 2018).

The OSMAC approach is convenient and effective, successfully activating a variety of bioactive SMs. The marine *Streptomyces* sp. HZP‐2216E could produce streptoarylpyrazinone A, a new derivative of *N*‐arylpyrazinone, when cultured in Gause's liquid medium (Zhang, Chen, Zhang, et al. 2017); while it induced the biosynthesis of the indolizinium alkaloid streptopertusacin A, which exhibited moderate anti‐MRSA (methicillin‐resistant *Staphylococcus aureus*) activity, when cultured on glucose‐yeast‐malt solid medium (Zhang, Chen, Chai, et al. 2017). Another marine *Streptomyces* sp. RKND004 was fermented in 14 different media, and combined with UPLC‐HRMS‐based metabolomics screening, led to the identification of Terrosamycins A and a novel derivative Terrosamycins B, both of which demonstrated strong activity against Gram‐positive bacteria and two breast cancer cell lines (Sproule et al. 2019).

In addition to alterations in medium composition, changes in culture conditions such as culture methods, temperature, salinity, and pH could also induce the production of new SMs. Six new antifungal polyene polyols were obtained from *Streptomyces* sp. CHQ‐64 under shaking culture, while a new hybrid isoprene alkaloid drimentine I was isolated under static culture (Che et al. 2016). Eighteen thermotolerant actinomycetes were cultivated in six different media at 30°C and 45°C, and 131 “heat shock metabolites” were identified by comparing the metabolic profiles of cultures at these two temperatures (Saito et al. 2020). Among of them, a novel SM of murecholamide from thermotolerant *Streptomyces* sp. AY2 was identified (Saito et al. 2020).

The addition of metal ions in medium activated the new SMs as well. Metal ion induction, also known as “metal stress,” refers to the application of specific concentrations of metal ions to the culture of strains. Heavy metals induce oxidative stress reactions to generate reactive oxygen species (ROS), leading to the formation of lipid hydroperoxides, and then converted to oxylipins, thereby activating the biosynthetic gene cluster of SMs (Auckloo et al. 2017). A new antimicrobial cyclic peptide NC‐1 was isolated from *Streptomyces* sp. FXJ1.172 on glucose‐yeast extract‐malt extract (GYM) medium containing iron ion (Liu et al. 2016). Nickel ion (NiCl_2_･6H_2_O) was identified as the most effective elicitor of stress in the marine *Streptomyces pratensis* NA‐ZhouS1, and activating the strain to produce two novel angucycline antibiotics of stremycin A and B. (Akhter et al. 2018).

Coculture with other microorganisms is another common cultivation‐based approach. In pure culture, the lack of stimulatory or signaling molecules involved in chemical communication and antagonism may result in BGCs silence (Moussa et al. 2019). However, cocultivation mimics the interactions within natural microbial communities, where the molecule secretion and cell‐to‐cell contact between microbes can prompt them to produce SMs that contribute to defense, communication, or competition (Zhang, He, et al. 2017). Coculture of marine *Streptomyces* sp. JB5 and *Bacillus* sp. GN1 led to the discovery of a new cyclic hexapeptide of dentigerumycin E which exhibited antiproliferative and antimetastatic activities against human cancer cells (Shin et al. 2018). A novel small‐molecule antibiotic amycomicin was isolated from the coculture of *Streptomyces coelicolor* M145 and *Amycolatopsis* sp. AA4, and amycomicin showed specific inhibition of *Staphylococcus aureus* (Pishchany et al. 2018).

In this study, the OSMAC method was employed to induce the production of SMs from marine *Streptomyces* sp. FJNU027. Using various media to culture FJNU027 strain, the differential product (4,4′,5,5′‐tetramethyl‐[1,1′‐biphenyl]‐2,2′‐diol) was isolated and structure determined through chromatography, LC‐MS and NMR. Besides, transcriptome assay was conducted to analyze the metabolic network of FJNU027 strain under different media conditions.

## 2 Materials and Methods

### 2.1 Strain and Media

The wild‐type of marine *Streptomyces* sp. FJNU027 strain (endowed by Xiamen Medical College of China) was used in this assay. The 20% YPD solid medium (4 g/L tryptone, 4 g/L glucose, 2 g/L yeast extract, 1.5 g/L agar) was used for the spore formation of strain FJNU027. The modified Gauze's medium (15 g/L soluble starch, 15 g/L glucose, 1 g/L KNO_3_, 0.5 g/L K_2_HPO_4_·3H_2_O, 0.2 g/L MgSO_4_·7H_2_O, 0.01 g/L FeSO_4_·7H_2_O), oligotrophic medium (5 mg/L soluble starch, 5 mg/L glucose, 1 g/L KNO_3_, 0.5 g/L K_2_HPO_4_·3H_2_O, 0.2 g/L MgSO_4_·7H_2_O, 0.01 g/L FeSO_4_·7H_2_O), eutrophic medium (15 g/L soluble starch, 15 g/L glucose, 1 g/L KNO_3_, 0.5 g/L K_2_HPO_4_·3H_2_O, 0.2 g/L MgSO_4_·7H_2_O, 0.01 g/L FeSO_4_·7H_2_O, 10 g/L tryptone, 2 g/L yeast extract), and rice medium (660 g/L rice) were prepared by sea water and used for the production of SMs in strain FJNU027.

### 2.2 Liquid Fermentation and Crude Extracts Preparation

The method for fermentation and crude extracts preparation was similar to previous study (Wang et al. 2023). In detail, the strain FJNU027 was cultured on 20% YPD solid medium, 28°C for 7 d. The mature spores were collected and cultured in 100 mL modified Gauze's medium, 28°C for 2 d with shaking at 220 rpm, and then 5% of above seed culture was transferred into 200 mL modified Gauze's medium or oligotrophic medium, 28°C for 20 d with shaking at 220 rpm. To prepare the crude extracts, the 200 mL culture was centrifuged (8000 rpm, 10 min), and the supernatant was treated twice with the equal volume of ethyl acetate. The organic phase was collected and dried by rotary evaporator.

### 2.3 HPLC Analysis of the Crude Extracts

The crude extracts were weighed and dissolved in methanol with the final concentration of 10 mg/mL, and then analyzed by HPLC using RP‐18 chromatography column (Thermo, Acclaim 120, 5 μm, 4.6 × 250 mm). The mobile phase consisted of solvents A (H_2_O, 0.1% formic acid) and B (acetonitrile, 0.1% formic acid). The gradient program was as follows: 5% B in A for 0 to 5 min, and increased to 80% B at 20 min, then to 100% B at 25 min, maintained to 30 min, and back to 5% B at 35 min. The flow rate was 1.0 mL/min with the UV detection of 254 nm.

### 2.4 Mass Fermentation, Isolation and Structure Determination of 1

The seed culture was prepared using the method described above, and then 5% of it was transferred into 30 L oligotrophic medium, 28°C for 20 d with shaking at 220 rpm. The 30 L culture was centrifuged (8000 rpm, 10 min), and the supernatant was treated twice with the equal volume of ethyl acetate. The ethyl acetate extract was dried using a rotavapor (Buchi, Rotavapor R‐200) to afford the crude extract (201.7 mg), which was subsequently isolated via column chromatography over Sephadex LH‐20 (MeOH) with the flow rate of 15 ~ 18 s/drop. The fractions were detected using HPLC and the fractions containing **1** were combined to yield a new fraction (2.9 mg). Finally, the new fraction was purified via HPLC to obtain compound **1** (1.4 mg). The pure compound **1** was dissolved in methanol‐*d*4, then the ^1^H‐NMR, ^13^C‐NMR, DEPT, ^1^H‐^1^HCOSY, HSQC, and HMBC spectra were acquired on a Bruker AVANCE NEO 600 spectrometer operating at 600/150 MHz. The molecular weight of compound **1** was determined through LC‐MS (Thermo, LCQ FLEET).

### 2.5 RNA Sequencing and Transcriptomic Analysis

RNA sequencing and transcriptomic analysis were performed using previous methods (Wang et al. 2023). The wild‐type of marine *Streptomyces* sp. FJNU027 strain was grown in modified Gauze's medium and oligotrophic medium at 28°C for 8 d. The mycelia of two media were collected by centrifugation (8000 rpm, 10 min). RNA was extracted via Total RNA Extractor (Trizol) kit (Sangon, China), then the RNA concentration and integrity were determined using Qubit 2.0 RNA detection kit (Life, United States) and agarose gel electrophoresis, respectively. To reduce sequencing interference, rRNA was removed by Ribo‐off rRNA depletion kit (Vazyme, China), and DNA was digested using DNase I (Vazyme, China). The fragmentation, reverse‐transcription, 3′ ends dA‐Tailing, Adapter ligation and DNA library construction of purified RNA were performed using VAHTSTM stranded mRNA‐seq library prep kit for illumina (Vazyme, China).

The DNA library was sequenced by illumine NovaSeq. 6000, and the raw data were cleaned and evaluated whether the sequencing data was suitable for subsequent analysis. Then the clean reads were compared to its own genome through BCFtools and Rockhopper. The expression levels were analyzed using featureCounts and WGCNA, and the differentially expressed genes were obtained by DESeq. 2 with a selection threshold of qValue < 0.05 and fold change ≥ 2.0. Genes and Genomes Ontology (GO) and Kyoto Encyclopedia of Genes and Genomes (KEGG) enrichment analysis were performed using topGO and clusterProfiler software, respectively.

## 3 Results

### 3.1 Activation of the Differential Secondary Metabolite in Oligotrophic Medium

**Figure 1:**
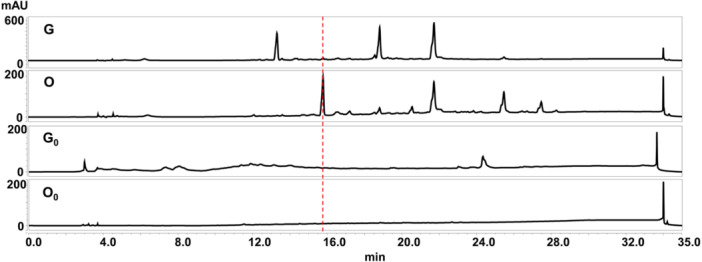
HPLC analysis of secondary metabolites from *Streptomyces* sp. FJNU027. G represented the FJNU027 strain cultured in modified Gauze's medium; O represented the FJNU027 strain cultured in oligotrophic medium; G_0_ and O_0_ represented modified Gauze's medium and oligotrophic medium without the FJNU027 strain, using as controls.

**Figure 2:**
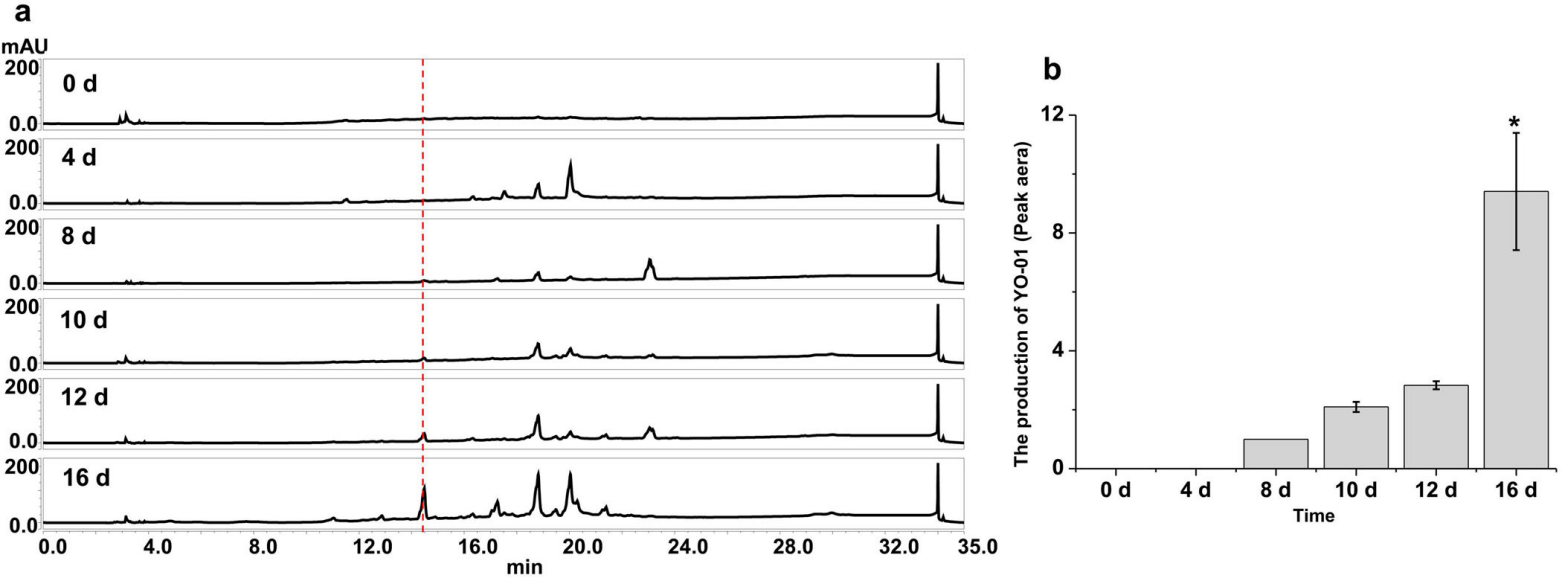
The production of **1** in oligotrophic medium. (a) HPLC analysis of **1** from FJNU027 strain cultured for 0 d, 4 d, 8 d, 10 d, 12 d and 16 d; (b) Quantification of **1** from these cultures.

To activate novel SMs from *Streptomyces* sp. FJNU027, OSMAC method was performed. The FJNU027 strain was fermented in modified Gauze's medium, oligotrophic, eutrophic and rice media. The crude organic extract was dissolved into methanol with a concentration of 10 mg/mL for HPLC analysis. The results showed that compared with the modified Gauze's medium, a distinct peak appeared at the retention time of 15.6 ~ 15.65 min under oligotrophic culture conditions (Figure 1). Additionally, the effect of fermentation time on differential product biosynthesis was analyzed. The crude extracts from cultures at 0 d, 4 d, 8 d, 10 d, 12 d and 16 d were analyzed by HPLC. The results showed the differential product appeared when the strain was cultured for 8 d, and the yield was gradually enhanced, as the culture time increased (Figure 2). However, the SMs from eutrophic and rice media showed no differences compared to those from control samples (Supporting Information S1: Figure S1).

### 3.2 Structure Determination of 1

**Figure 3:**
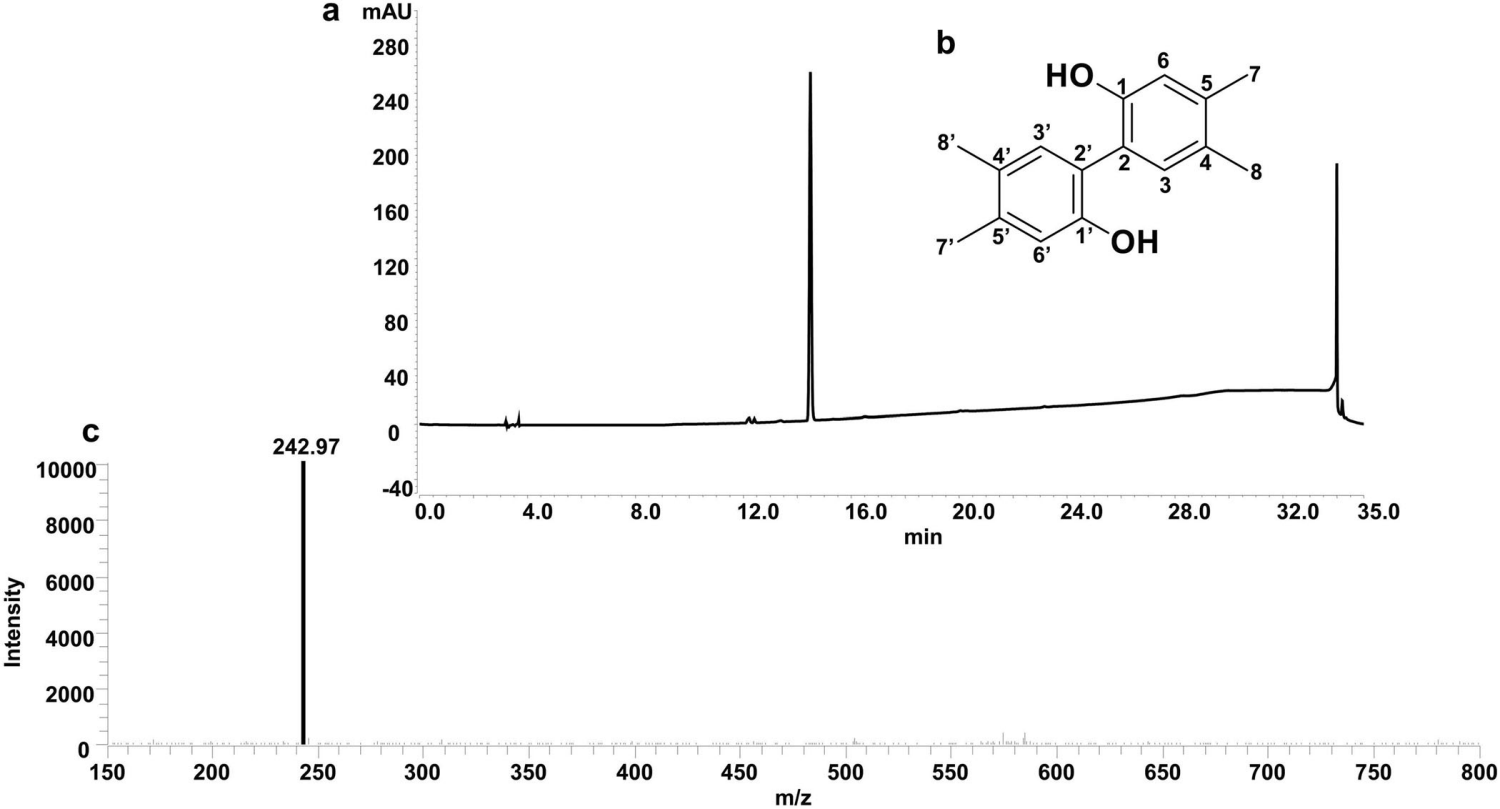
The structure determination of **1**. HPLC analysis (a) and the structure (b) of the pure **1**; (c) LC‐MS analysis of **1**.

To determine the structure of differential product from the oligotrophic medium, the culture of the FJNU027 strain was scaled up, yielding 2 mg of the compound (light yellow powder) from 30 L of culture (Figure 3a). The purified compound was then analyzed by spectroscopic methods. In the ^1^H‐NMR spectrum (600 MHz, DMSO‐*d*6), the compound gave δ 7.86 (1H, s, H‐3/3′), 7.65 (1H, s, H‐6/6′), 2.47 (3H, s, H‐7/7′), 2.44 (3H, s, H‐8/8′) (Figure S2). The ^13^C‐NMR spectrum showed resonances at δ 165.5 (C‐1/1′), 142.0 (C‐2/2′), 128.9(C‐3/3′), 136.7 (C‐4/4′), 145.9 (C‐5/5′), 125.8 (C‐6/6′), 20.2 (C‐7/7′), 19.6 (C‐8/8′) (Figure S3). In the heteronuclear multiple bond correlation (HMBC)experiment, the correlations of H_3_‐7 to C‐4/5/6, H_3_‐8 to C‐3/4/5, H‐3 to C‐2/5/6, and H‐6 to C‐3/4, together with their chemical shift of C‐1 to C‐8 (Supporting Information S1: Figure S4–S7), indicated the establishment of the unit of 3,4‐dimethylphenol. Comparison of the ^1^H NMR data those reported for the dimer of 3,4‐dimethylphenol revealed high similarity (Navarra et al. 2010). Therefore, the compound can be identified as the structure of 4,4′,5,5′‐tetramethyl‐[1,1′‐biphenyl]‐2,2′‐diol, which is the dimer of 3,4‐dimethylphenol (Figure 3b) and confirmed by ESI‐MS data with an *m/z* 242.97 of [M + H]^+^ (C_16_H_18_O_2_) (Figure 3c).

### 3.3 Transcriptomics Analysis

The differential product was activated in oligotrophic medium, which indicated its metabolic network was changed when compared to that of modified Gauze's medium. Therefore, the transcriptomic analysis of FJNU027 strain cultured on oligotrophic medium (O group) and modified Gauze's medium (G group) were carried out. In total, 51,599,354 and 46,678,424 clean reads were obtained from O group and G group, respectively. The average read length was 136.84 bp of O group and 131.62 bp of G group, besides, 99.10% and 87.29% of clean reads were mapped in O group and G group, respectively. Among them, 3874 differentially expressed genes (DEGs) were identified, including 914 up‐ and 2960 downregulated genes, and 374 DEGs specifically expressed in oligotrophic condition, while 175DEGs specifically expressed in normal condition (Figure S8).

**Figure 4:**
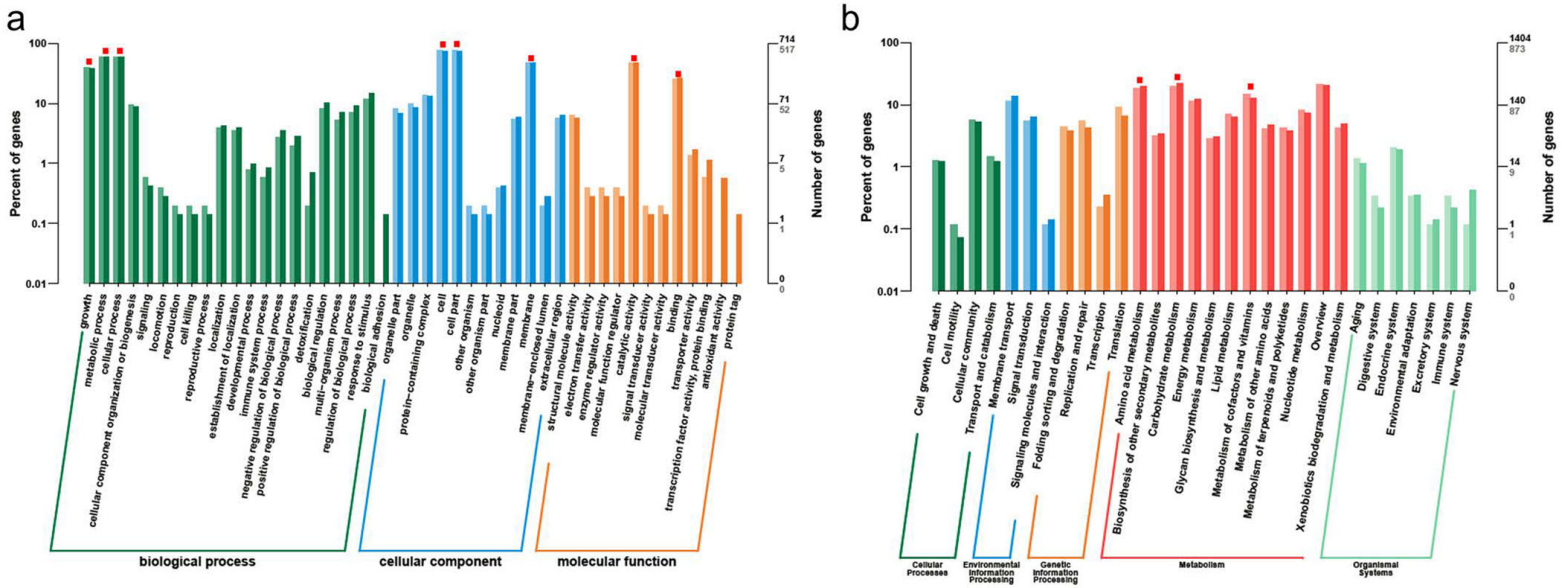
The GO analysis (a) and KEGG analysis (b) of DEGs. The red points represented the main subcategories in each category. The dark columns represented all genes involved in subcategories, the light columns represented the DEGs in subcategories.

GO analysis showed that 1115, 1255, 428 DGEs were enriched in biological process, cellular component and molecular function, respectively (Figure 4a). The main categories were growth (205 DEGs), metabolic process (308 DEGs) and cellular process (308 DEGs) in biological process; cell (391 DEGs), cell part (391 DEGs) and membrane (247 DEGs) in cellular component; catalytic activity (247 DEGs) and binding (130 DEGs) in molecular function (Figure 4a). KEGG analysis revealed that the identified DEGs were mainly enriched in metabolism pathway (1040 DEGs), as well as cellular processes (74 DEGs), environmental information processing (149 DEGs), genetic information processing (166 DEGs), and organismal systems (41 DEGs) (Figure 4b). In metabolism pathway, the DEGs were mainly associated with carbohydrate metabolism (170 DEGs), amino acid metabolism (162 DEGs) and metabolism of cofactors and vitamins (129 DEGs) (Figure 4b).

The highly significant DEGs (10 up‐ and 10 downregulated genes) were selected with threshold of TPM (Transcripts Per Million) > 100 (Supporting Information S1: Tables S1 and S2). Among 10 highest upregulated genes, three of them encoding glycoside hydrolase which involved in utilization of carbohydrate, two of them encoding peptidase, and the other half of genes encoding regulator, ribonuclease, hydrolase, RNA polymerase sigma factor and peptidase inhibitor, respectively. Besides, among 10 highest downregulated genes, three of them were transporter genes, two regulator genes, two of them encoding succinate dehydrogenase, and other three genes encoding 1‐phosphofructokinase, *N*‐formylglutamate amidohydrolase and FAD‐binding oxidoreductase, respectively.

## 4 Discussion

In this study, the SM of 3, 4‐dimethylphenol dimer was identified from *Streptomyces* sp. FJNU027 using OSMAC approach. This compound was only reported in the oxidation of 3, 4‐dimethylphenol by laccase in vitro (Navarra et al. 2010), making this the first report of its isolation from an organism. To explore the potential biosynthesis mechanism of 3, 4‐dimethylphenol dimer, the laccase‐like genes (FZ01GL006124) from FJNU027 strain were found using the whole protein sequences to blastp with a known laccase from *Streptomyces coelicolor* (Sherif et al. 2013). However, this gene was poorly expressed in both O group and G group according to the transcriptome data. Moreover, 3, 4‐dimethylphenol was added into modified Gauze's medium to check whether FJNU027 strain could convert it to its dimer form. Unexpectedly, no 3, 4‐dimethylphenol dimer was detected (Supporting Information S1: Figure S9). However, the recovered production of 3, 4‐dimethylphenol from modified Gauze's medium without FJNU027 strain was significantly decreased, indicating it was unstable and might be degraded spontaneously (Supporting Information S1: Figure S10). Besides, no 3, 4‐dimethylphenol was detected during the growth of FJNU027 strain in oligotrophic medium (Figure S11). Nonetheless, further studies might be performed to elucidate the biosynthesis of 3, 4‐dimethylphenol dimer, such as the deletion of laccase‐like gene and evaluating the production in the mutant, and purification of laccase‐like protein to carry out in vitro reaction with 3, 4‐dimethylphenol. A number of aromatic SMs are biosynthesized using polyketide synthases (PKSs) in actinomycetes (Zhang, Pan, et al. 2017). Transcriptomic data showed that the expression of one PKS gene (FZ01GL006410) was significantly enhanced in O group (Supporting Information S1: Table S3). Amino acid sequence analysis of the PKS revealed a Methyltransferase domain and a TPL (tyrosine phenol‐lyase)‐like domain which was responsible for phenol formation (Kim et al. 2014) (Supporting Information S1: Figures S12 and S13). Moreover, an adjacent gene (FZ01GL006417) encoding cytochrome P450 also exhibited higher expression in O group (Supporting Information S1: Table S3). It was found the cytochrome P450 catalyzed benzene formation in the biosynthesis of some aromatic polyketides (Yang et al. 2023). Together, these findings suggested that the TPL‐like domain of PKS catalyzed tyrosine to form phenol which was subsequently methylated by Methyltransferase domain of PKS to generate 3, 4‐dimethylphenol, and then cytochrome P450 converted 3, 4‐dimethylphenol to its dimer form. As we known, the biological function of 3, 4‐dimethylphenol and its dimer form had not been explored, however, the natural phenolic compounds exhibited antioxidant or antimicrobial activity in their producing strains (Alfieri et al. 2022; Chen et al. 2024). Therefore, FJNU027 strain might produce the dimer of 3, 4‐dimethylphenol with antioxidant and/or antimicrobial activity to gain competitive growth advantage under harsh conditions. Among 10 highest upregulated genes, three of them are glycoside hydrolase genes which were barely expressed in modified Gauze's medium, it might be due to the relief of glucose effect in oligotrophic medium. The extremely low concentration of glucose was easily consumed and then the expression of glycoside hydrolases was activated in oligotrophic medium. Oppositely, three genes (one phosphofructokinase and two succinate dehydrogenase) involved in glycolysis pathway and TCA cycle were highest downregulated in O group. The weak growth of FJNU027 strain in oligotrophic medium might be the reason for low expression of these genes. The transcriptome data exhibited the significant changes in expression profile of FJNU027 strain cultured on different media, which led to the change of metabolic profile in FJNU027 strain.

## 5 Conclusions

Marine *Streptomyces* are the promising source for discovery of lead compounds. In this study, the oligotrophic condition was used to stimulate the FJNU027 strain producing 3, 4‐dimethylphenol dimer which was firstly isolated from organisms. The results suggested the OSMAC approach is an effective way for novel SMs discovery from *Streptomyces*. Furthermore, the induction mechanism of 3, 4‐dimethylphenol dimer should be explored in future studies.

## Author Contributions

**Feifei Wang:** conceptualization, methodology. **Huimin Yuan:** methodology. **Cuie Bai:** methodology. **Haiyan Li:** methodology. **Li Xu:** writing – review and editing. **Lingjun Yu:** supervision, writing – original draft. **Lianzhong Luo:** writing – review and editing, funding acquisition. **Yongbiao Zheng:** supervision, writing – review and editing, funding acquisition.

## Ethics Statement

The authors have nothing to report.

## Conflicts of Interest

The authors declare no conflicts of interest.

## Supporting information


**Table S1:** The ten highest upregulated genes from transcriptome data. **Table S2:** The ten highest downregulated genes from transcriptome data. **Table S3:** The transcription of the PKS gene and P450 gene from transcriptome data.

## Data Availability

The transcriptome sequence data are openly available in the GEO database under accession GSE278152: https://www.ncbi.nlm.nih.gov/geo/query/acc.cgi?&acc=GSE278152. Other data are included within the article and its Supporting Information.
