# Supplementary material for: Activation of the Dimer of 3, 4‐dimethylphenol Production From Marine Streptomyces sp. FJNU027 Under Oligotrophic Condition
Source: Microbiologyopen. 2025 Dec 1;14(6):e70191. doi: 10.1002/mbo3.70191 (PMC12669078; doi:10.1002/mbo3.70191)
Supplement: Supplementary file 1 — Table S1: The ten highest upregulated genes from transcriptome data. Table S2: The ten highest downregulated genes from transcriptome data. Table S3: The transcription of the PKS gene and P450 gene from transcriptome data. [file MBO3-14-e70191-s001.docx]

**Activation of the dimer of 3, 4-dimethylphenol production from marine *Streptomyces* sp. FJNU027 under** **oligotrophic condition**

Feifei Wang^1^, Huimin Yuan^1^, Cuie Bai^1^, Haiyan Li^1^, Li Xu^2^, Lingjun Yu^1,^*, Lianzhong Luo^2,^*, Yongbiao Zheng^1,^ *

^1^School of Life Sciences, Fujian Normal University, Fuzhou 350117, China.

^2^Engineering Research Center of Marine Biopharmaceutical Resource, Xiamen Medical College, Xiamen 361023, China

* Correspondences: yulj113@fjnu.edu.cn; lianzhongluo@foxmail.com; yongbiaozheng@fjnu.edu.cn


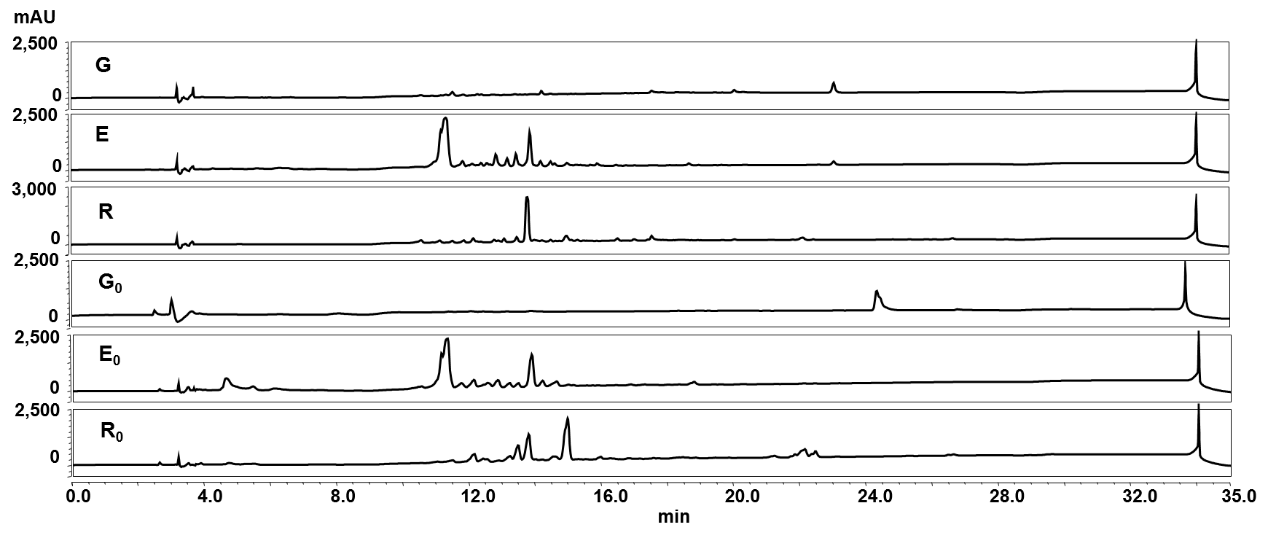


**Figure S1** HPLC analysis of secondary metabolites from *Streptomyces* sp. FJNU027. G represented the FJNU027 strain cultured in modified Gauze's medium; E represented the FJNU027 strain cultured in eutrophic medium; R represented the FJNU027 strain cultured in rice medium; G_0_, E_0_ and R_0_ represented modified Gauze's medium, eutrophic medium and rice medium without the FJNU027 strain, using as controls.


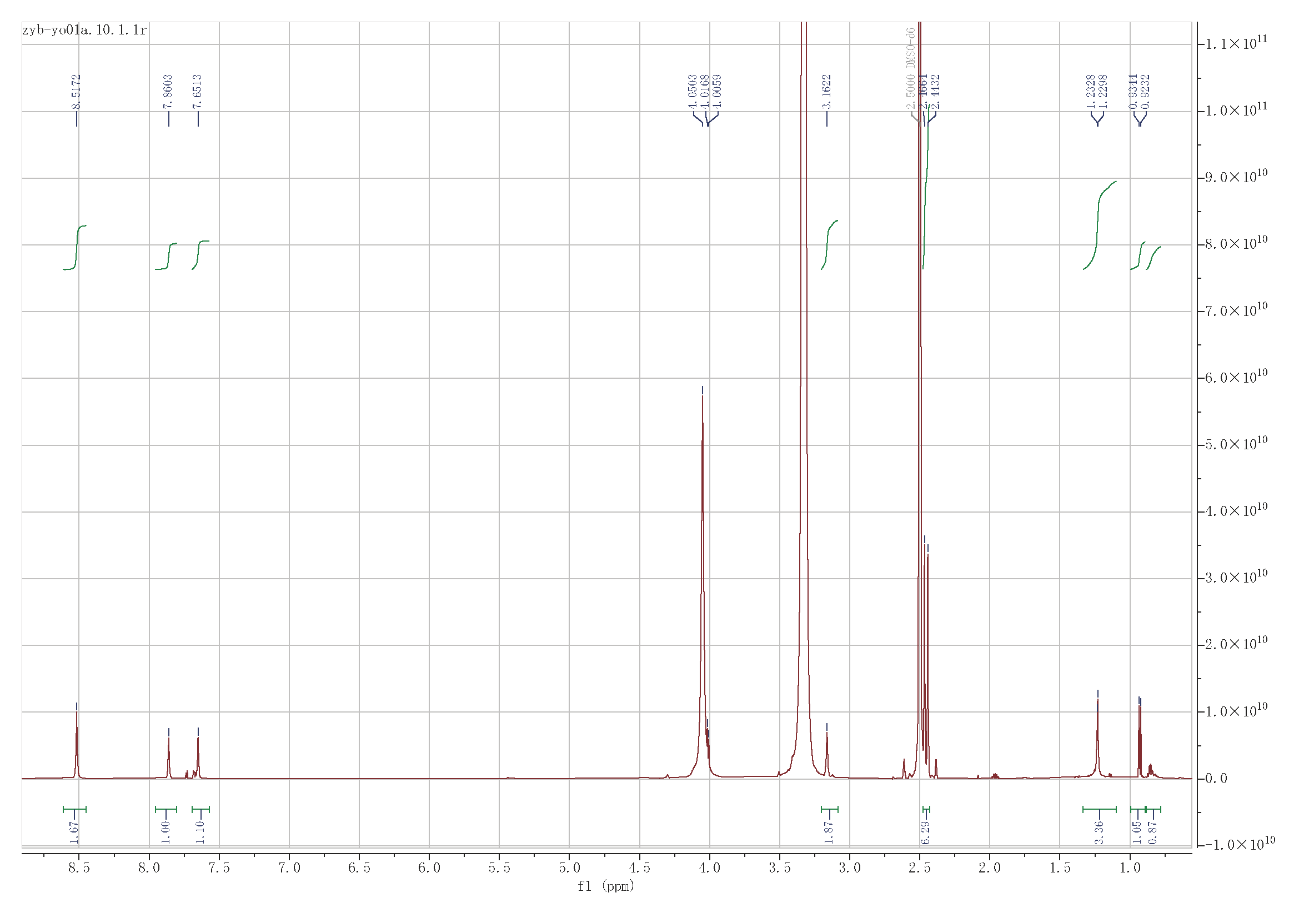


**Figure S2** ^1^H-NMR spectra of Compound **1**


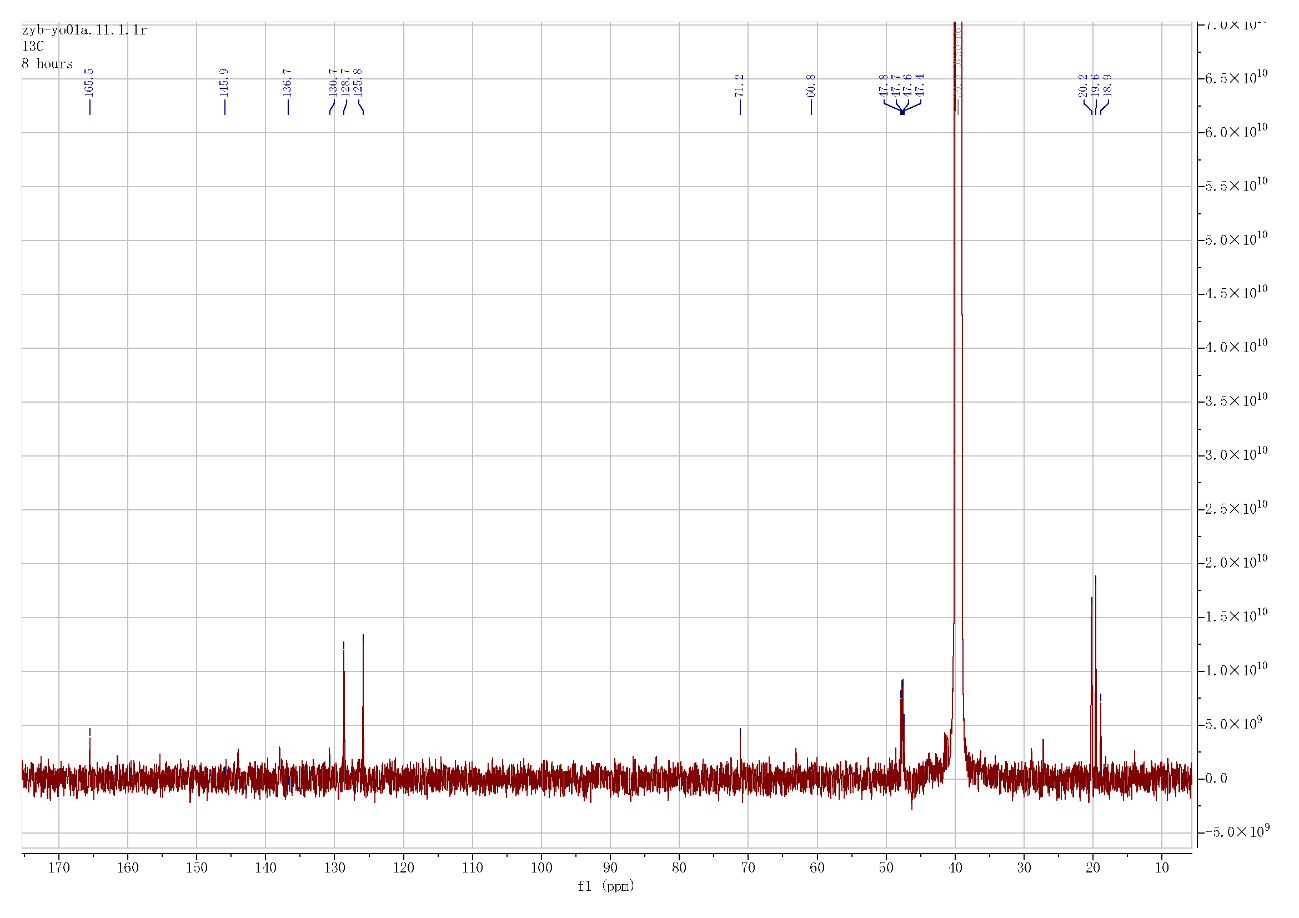


**Figure S3** ^13^C-NMR spectra of Compound **1**


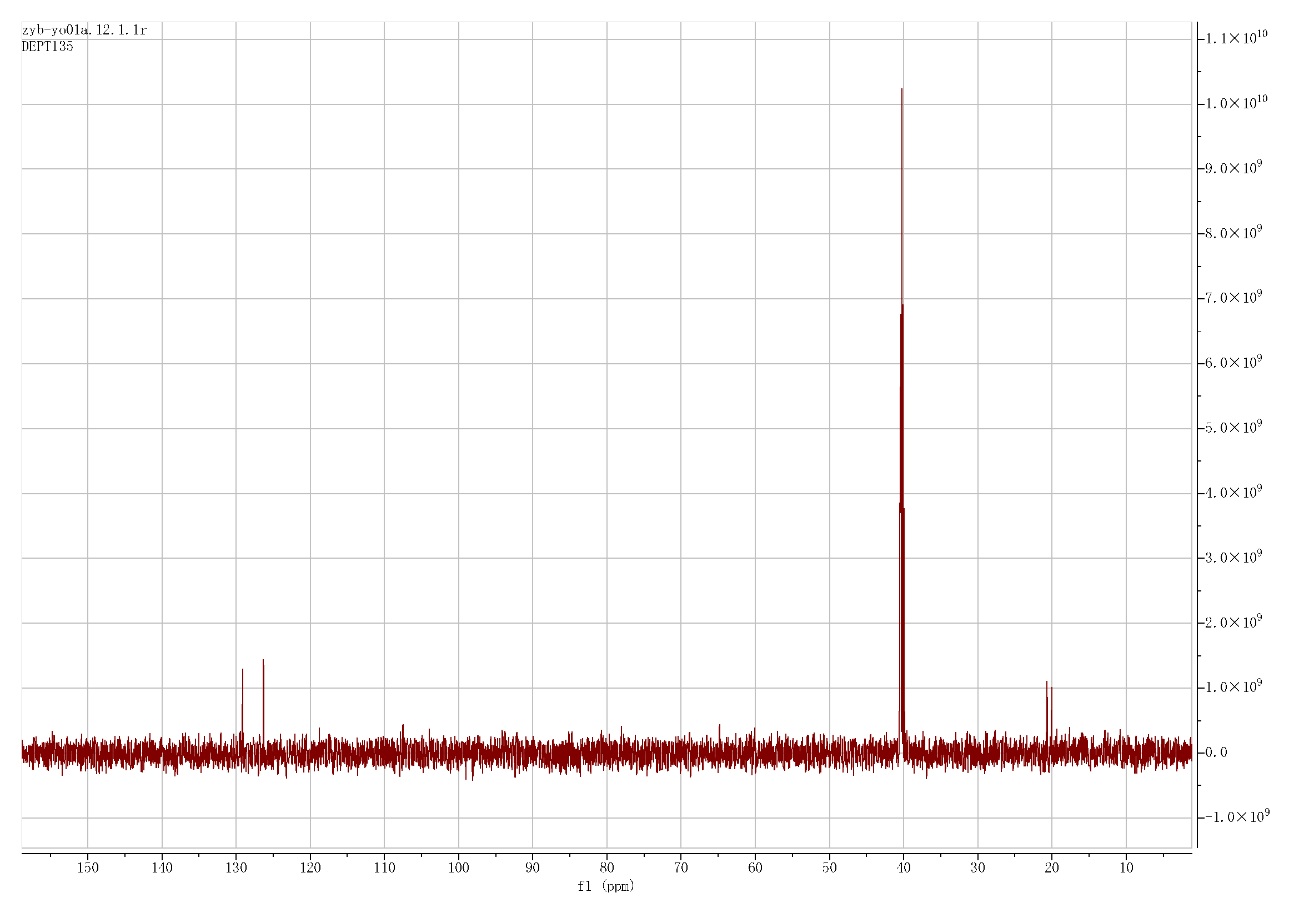


**Figure S4** DEPT spectra of Compound **1**


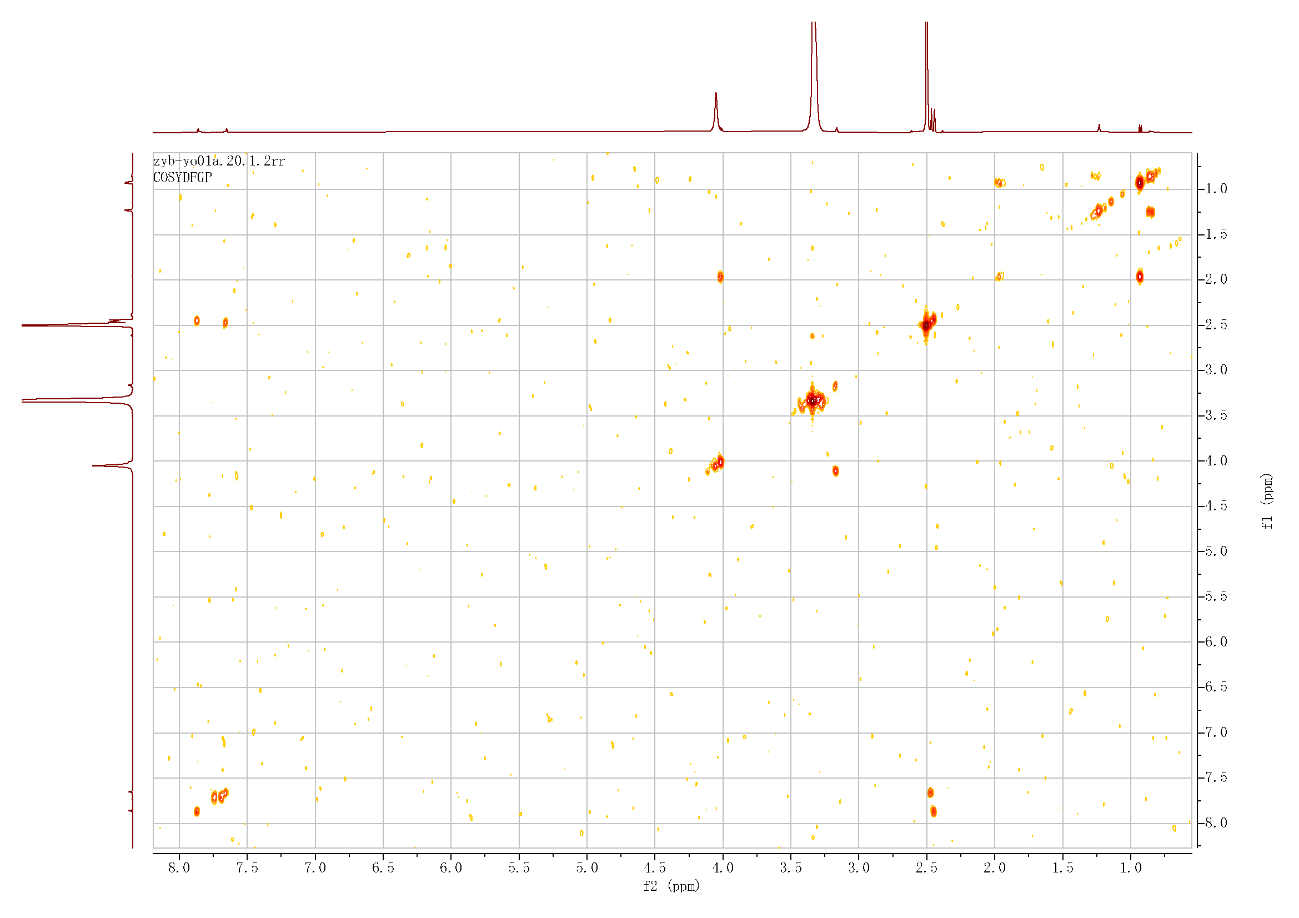


**Figure S5** ^1^H-^1^HCOSY spectra of Compound **1**


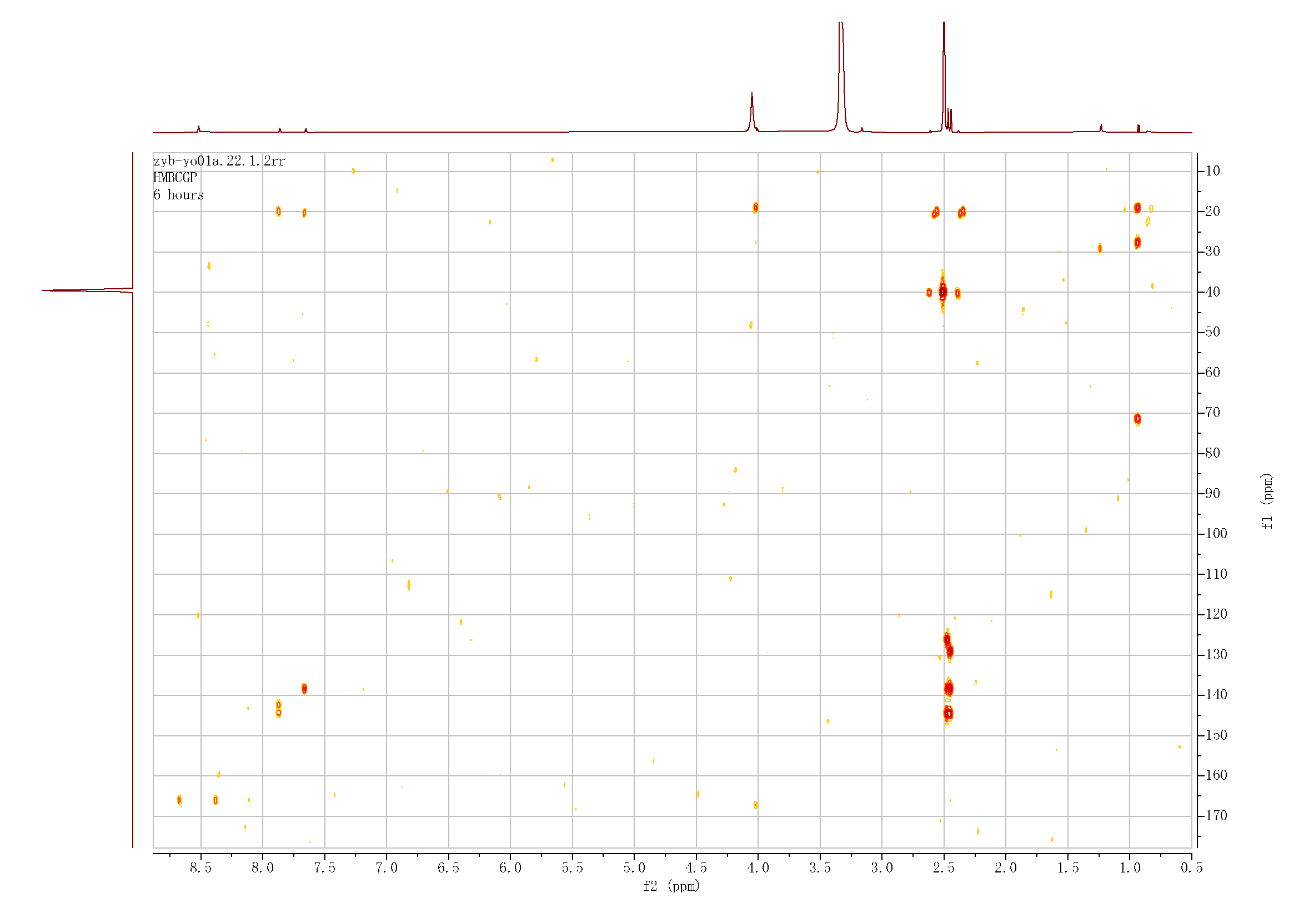


**Figure S6** HMBC spectra of Compound **1**


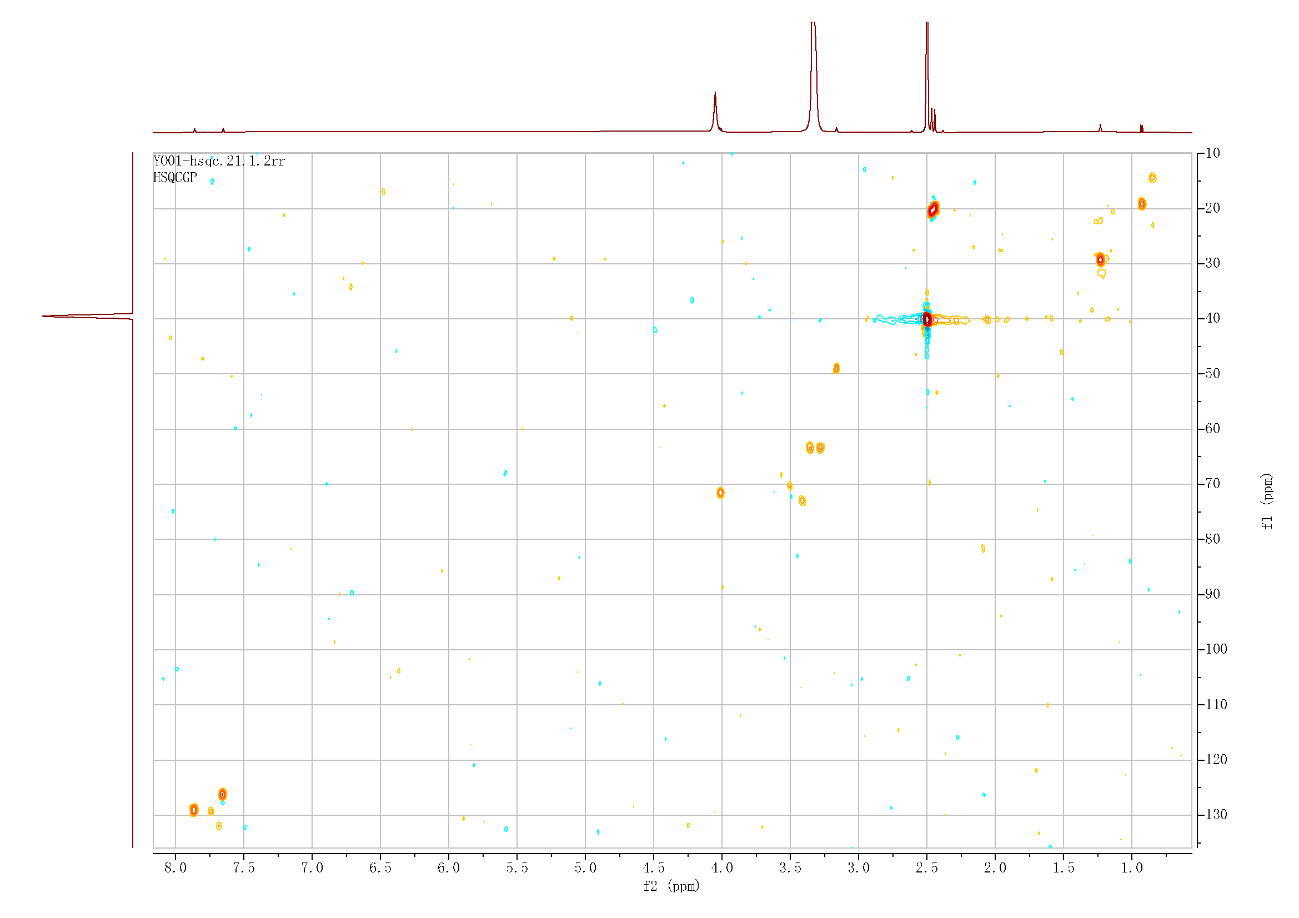


**Figure S7** HSQC spectra of Compound **1**


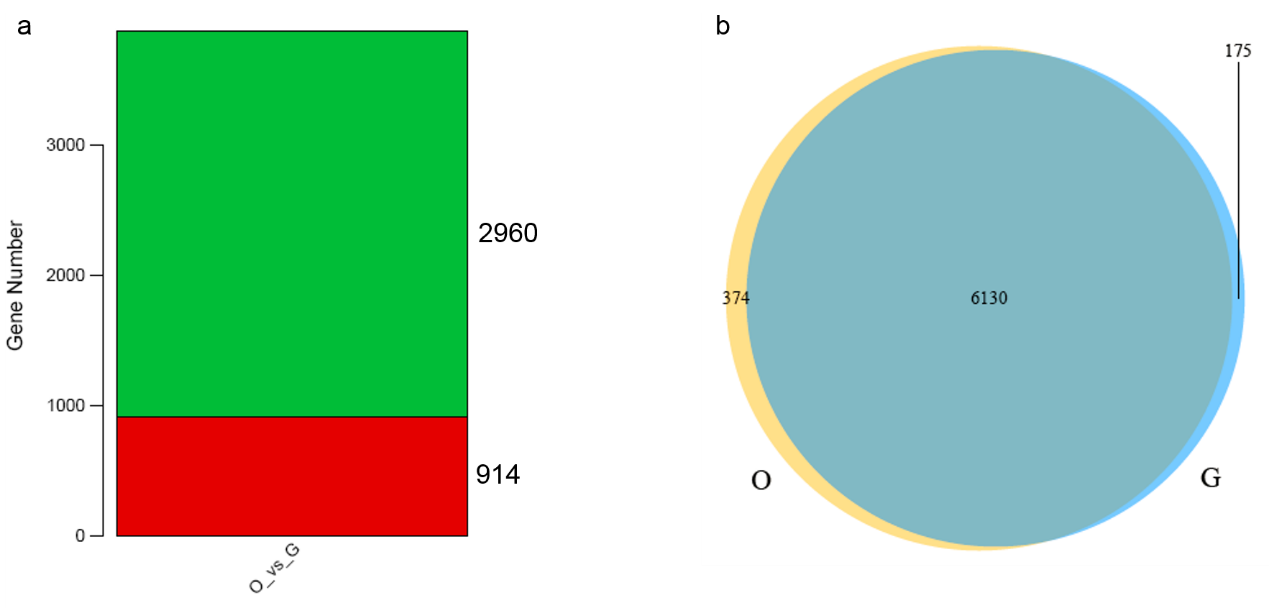


**Figure S8** The differentially expressed genes (DEGs) between O group and G group. (a) The barplot of DEGs, the red barplot represented upregulated genes and the green barplot represented downregulated genes; (b) The venn map of gene expression, the overlapping area represented genes expression in both groups; the non-overlapping area represented the specific genes expression between different groups. O represented the sample from oligotrophic medium; G represented the sample from modified Gauze's medium.


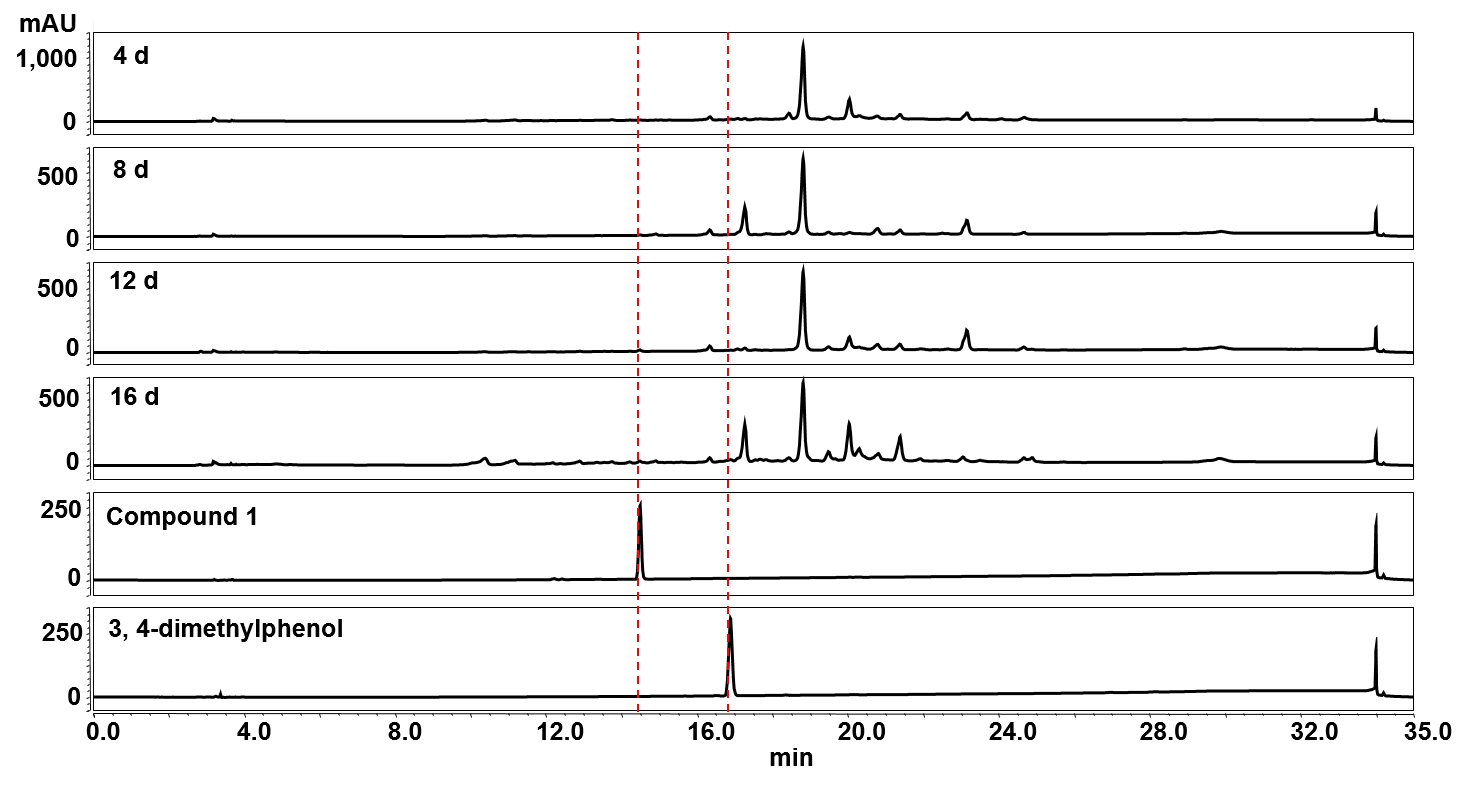


**Figure S9** HPLC analysis of the **1** production. The FJNU027 strain was cultured in modified Gauze's medium with the exogenous addition of 3, 4-dimethylphenol (final concentration was 80 mM) for 4 d, 8 d, 12 d, and 16 d. Compound **1** and 3,4-dimethylphenol were used as standards.


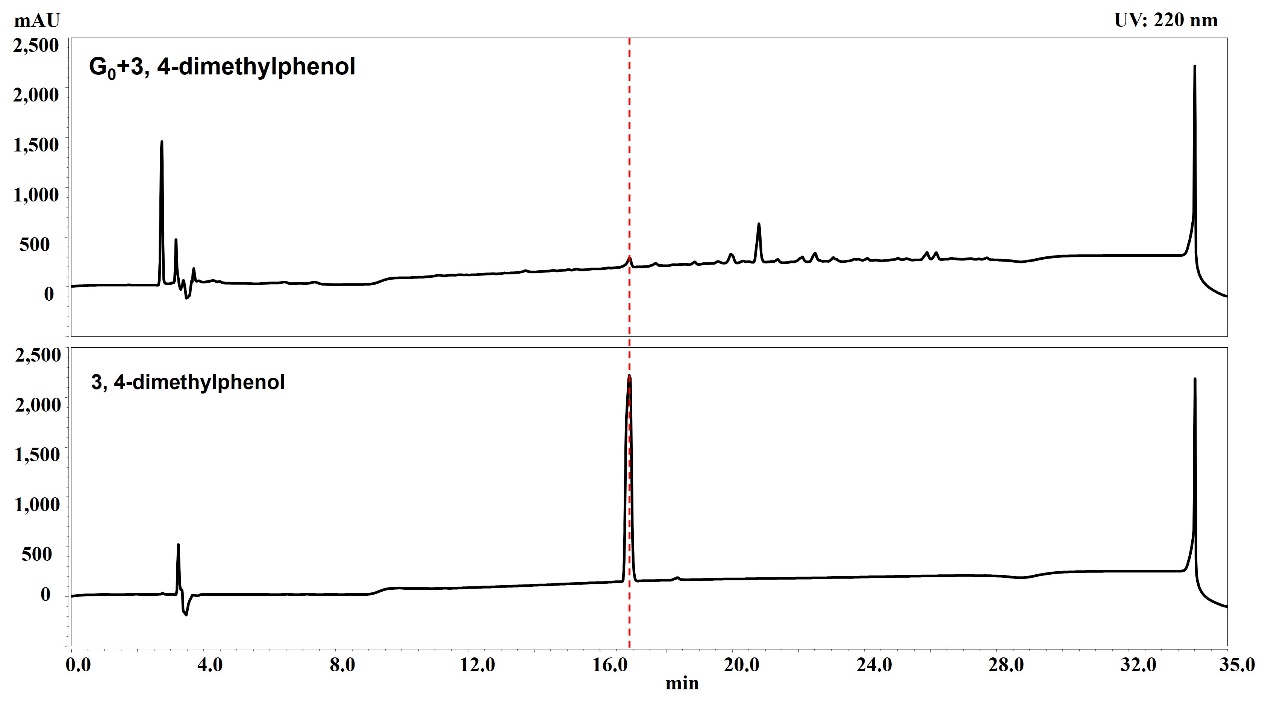


**Figure S10** HPLC analysis of 3,4-dimethylphenol. G_0_+3,4-dimethylphenol represented 3, 4-dimethylphenol was added into modified Gauze's medium without the FJNU027 strain (final concentration was 80 mM) for 4 d. The same concentration of pure 3,4-dimethylphenol was used as the standard.


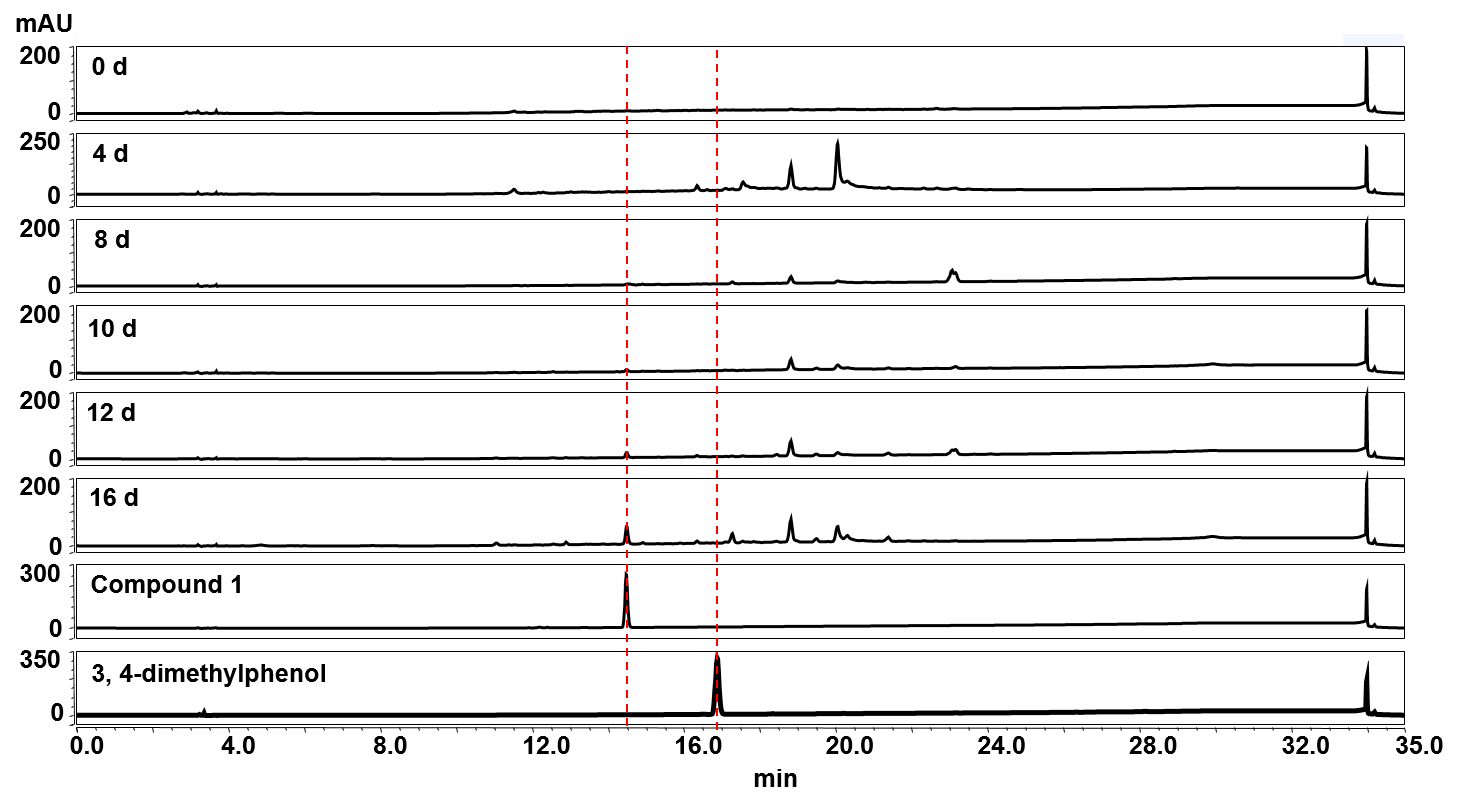


**Figure S11** HPLC analysis of the 3, 4-dimethylphenol production in oligotrophic medium. Compound **1** and 3,4-dimethylphenol were used as standards.


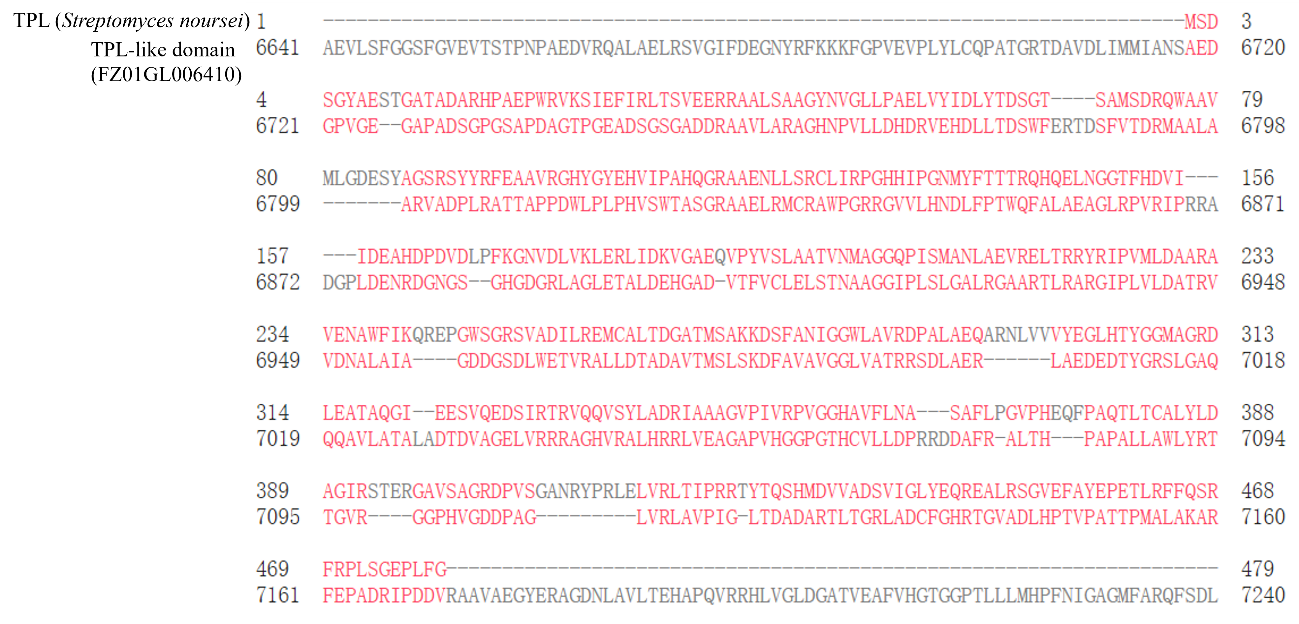


**Figure S12** Alignment of the amino acid sequence of TPL (tyrosine phenol-lyase)-like domain in protein FZ01GL006410 with a known TPL from *Streptomyces noursei* (NCBI accession no. AIA01240.1). The red color indicates high conserved ones.


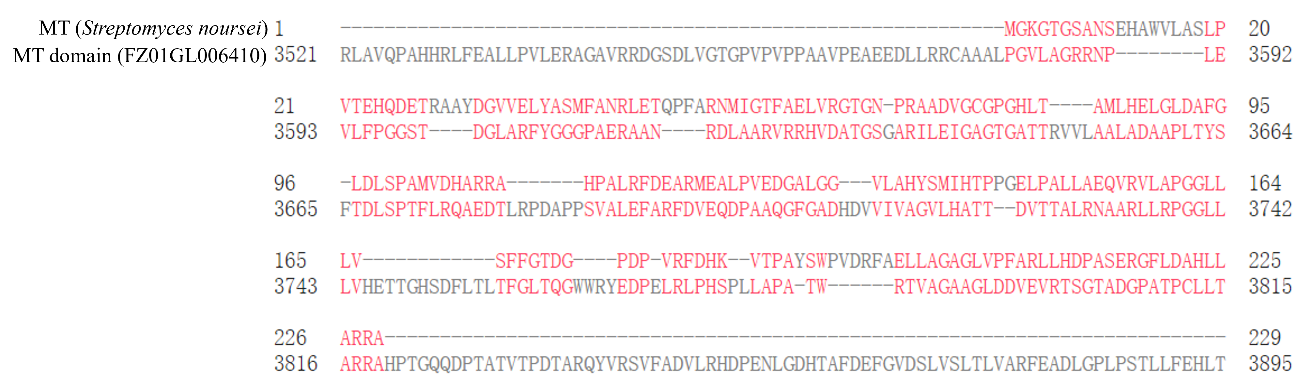


**Figure S13** Alignment of the amino acid sequence of MT (Methyltransferase) domain in protein FZ01GL006410 with a known MT from *Streptomyces noursei* (NCBI accession no. GGX01291.1). The red color indicates high conserved ones.

Table S1 The ten highest upregulated genes from transcriptome data

| Gene ID | log_2_Fold Change (O/G) | qValue | Definition |
| --- | --- | --- | --- |
| FZ01GL002070 | 21.6 | 5.61E-291 | glycosidase |
| FZ01GL006260 | 21.1 | 2.11E-84 | TraR/DksA C4-type zinc finger protein |
| FZ01GL005775 | 20.3 | 1.35E-42 | ribonuclease |
| FZ01GL006040 | 8.6 | 0 | glycoside hydrolase |
| FZ01GL000546 | 7.4 | 0 | S8 family peptidase |
| FZ01GL004528 | 7.1 | 9.81E-164 | M14 family metallopeptidase |
| FZ01GL001555 | 7.1 | 1.97E-156 | SGNH/GDSL hydrolase family protein |
| FZ01GL002300 | 7.0 | 0 | RNA polymerase sigma factor |
| FZ01GL003984 | 6.4 | 0 | peptidase inhibitor family I36 protein |
| FZ01GL006790 | 6.1 | 2.74E-300 | glycoside hydrolase family 64 protein |

Table S2 The ten highest downregulated genes from transcriptome data

| Gene ID | log_2_Fold Change (O/G) | qValue | Definition |
| --- | --- | --- | --- |
| FZ01GL003426 | -22.1 | 1.99E-101 | MarR family transcriptional regulator |
| FZ01GL003427 | -10.0 | 0 | MFS transporter |
| FZ01GL004441 | -8.7 | 0 | MFS transporter |
| FZ01GL003026 | -8.1 | 1.61E-71 | 1-phosphofructokinase |
| FZ01GL006504 | -7.1 | 0 | fumarate reductase/succinate dehydrogenase |
| FZ01GL001953 | -7.1 | 4.18E-75 | ABC transporter |
| FZ01GL006505 | -7.1 | 0 | succinate dehydrogenase/fumarate reductase iron-sulfur subunit |
| FZ01GL003025 | -6.9 | 6.34E-103 | DeoR/GlpR family DNA-binding transcription regulator |
| FZ01GL006250 | -6.6 | 9.30E-82 | N-formylglutamate amidohydrolase |
| FZ01GL002088 | -6.5 | 0 | FAD-binding oxidoreductase |

Table S3 The transcription of the PKS gene and P450 gene from transcriptome data

| Gene ID | Mean TPM (O) | Mean TPM (G) |
| --- | --- | --- |
| FZ01GL006410 | 10.85 | 1.16 |
| FZ01GL006417 | 123.85 | 12.11 |
